# Unequal Returns: Education Fails to Fully Prepare Black and Latino Americans for Retirement

**DOI:** 10.31586/ojer.2024.1104

**Published:** 2024-11-21

**Authors:** Shervin Assari, Babak Najand, Hossein Zare, Amanda Sonnega

**Affiliations:** 1 Marginalized-Related Diminished Returns (MDRs) Research Center, Los Angeles, CA, USA; 2 Department of Family Medicine, Charles R. Drew University of Medicine and Science, Los Angeles, CA, USA; 3 Department of Urban Public Health, Charles R. Drew University of Medicine and Science, Los Angeles, CA, USA; 4 Department of Health Policy and Management, Johns Hopkins Bloomberg School of Public Health, Baltimore, MD, USA; 5 School of Business, University of Maryland Global Campus (UMGC), Adelphi, MD, USA; 6 Institute for Social Research, University of Michigan, Ann Arbor, CA, USA

**Keywords:** Retirement Preparedness, Educational Attainment, Minorities’ Diminished Returns, Racial Disparities, Latino, Black, Understanding America Study (UAS)

## Abstract

**Background:**

Retirement is a universal life stage, marking the culmination of an individual’s working years. However, many people face financial challenges during retirement due to insufficient financial planning. Retirement preparedness is essential for ensuring economic security and maintaining a high quality of life in later years. Education is often viewed as a key driver of retirement preparedness, as it is linked to higher earnings, better financial literacy, and improved decision-making. However, the Minorities’ Diminished Returns (MDRs) theory suggests that the economic, cognitive, and behavioral benefits of education are weaker for racial and ethnic minorities compared to non-Latino Whites.

**Objective:**

This study aims to examine the relationship between educational attainment and retirement preparedness, focusing on whether this association differs among Black, Latino, and non-Latino White individuals, using data from the Understanding America Study (UAS).

**Methods:**

Data were drawn from the UAS, a nationally representative internet-based panel survey. The sample included participants from diverse racial and ethnic backgrounds. Linear regression models were used to evaluate the association between educational attainment, measured in years of schooling, and retirement preparedness. Interaction terms were included to test whether the association varied by race and ethnicity. Models were adjusted for potential confounders, including age, sex, marital status, employment status, and immigration.

**Results:**

In the overall sample, higher educational attainment was significantly and positively associated with better retirement preparedness (p < 0.001). However, consistent with the MDRs framework, the strength of this association was significantly weaker for Black and Latino participants compared to non-Latino White participants (p < 0.05). Non-Latino Whites with higher education levels reported substantially better retirement preparedness, while the same level of education yielded smaller gains in retirement preparedness for Black and Latino individuals.

**Conclusion:**

The findings support the Minorities’ Diminished Returns theory, showing that although educational attainment enhances retirement preparedness for all groups, Black and Latino individuals derive fewer benefits compared to their non-Latino White counterparts. These disparities point to persistent structural inequalities and systemic barriers within the education system and labor market, as well as the effects of segregation and discrimination, which undermine the economic benefits of education for marginalized populations. Addressing these disparities requires targeted policy interventions aimed at eliminating racial and ethnic inequalities in retirement outcomes and ensuring equitable benefits from educational attainment for all groups.

## 1. Introduction

Retirement is a critical and often challenging life stage for older adults [1]. The transition to retirement marks a significant shift in an individual’s financial stability, requiring careful planning and preparation that should begin decades earlier [2]. Unfortunately, many individuals enter retirement without adequate financial support, largely due to a lack of financial preparedness, which can significantly impact their quality of life during this stage [3]. Among the various factors influencing a successful and fulfilling retirement, financial preparedness is one of the most important determinants, shaping how individuals experience retirement both economically and financially [4]. While wealth, inheritance, savings, pensions, and other assets contribute to retirement success, early financial planning and preparedness remain key predictors of a high-quality retirement [5].

Educational attainment plays a vital role in enhancing retirement preparedness [6]. Individuals with higher levels of education typically demonstrate greater financial preparedness for retirement [7, 8]. This is partly because educational attainment is linked to better financial literacy, numeracy, and decision-making skills [9]. Additionally, higher education often leads to more stable career opportunities and access to resources, such as financial planning programs and occupational benefits, which further enhance retirement preparedness [10, 11]. In contrast, those with lower levels of education face greater challenges in preparing for retirement, often resulting in financial insecurity and increased stress later in life [12, 13].

However, while education is generally associated with higher income and better employment opportunities, research shows that Black and Latino individuals often receive fewer financial benefits from education compared to their White counterparts [14–18]. For instance, even at similar levels of education, Black and Latino individuals tend to face greater occupational and life stress, earn lower wages, work in more precarious jobs, and experience harsher occupational expectations [19–21]. According to the theory of Minorities’ Diminished Returns (MDRs), the positive effects of education on various economic and behavioral outcomes are often less pronounced for marginalized racial and ethnic minorities [14, 22–33]. This pattern also extends to other areas, such as health outcomes and wealth accumulation, where racial and ethnic minorities often experience reduced returns from their educational achievements due to structural inequalities, labor market discrimination, and limited access to high-quality resources [34].

Building on the MDRs framework [34]—particularly in the context of life course, retirement, and occupational experiences [19–21]—this study aims to examine the effects of educational attainment on retirement preparedness and explore how these effects vary by race and ethnicity. Specifically, we hypothesize that while higher levels of education will generally be associated with greater retirement preparedness, this relationship will be weaker for racial and ethnic minorities, reflecting the diminished returns that these groups experience.

## 2. Methods

The Understanding America Study (UAS) [35–39] is a large, nationally representative, internet-based survey conducted by the University of Southern California (USC). The UAS is designed to gather extensive insights on a wide array of social, economic, and health-related issues across the U.S. population. The study employs probability-based sampling methods, drawing from post-office delivery sequence files to recruit participants. To ensure full inclusivity and representativeness, individuals who do not have internet access are provided with internet-enabled devices, such as tablets, along with internet services, enabling them to participate in the surveys.

The UAS panel includes about 10,000 participants, with nearly 5,000 individuals aged 50 and older. The UAS collects detailed data on numerous domains, including well-being, retirement readiness, cognitive functioning, health behaviors, and personality traits. These surveys are administered regularly—either annually or biennially—to capture longitudinal data on participants. In addition to socioeconomic and behavioral variables, UAS includes health-related metrics to provide continuous assessments of the participants’ health status over time.

The UAS [35–39] provides a rich dataset for examining the relationship between educational attainment and retirement preparedness, particularly when investigating variations by race and ethnicity. Its comprehensive data collection process allows for the exploration of how different demographic factors, such as age and employment, may moderate the association between education and financial readiness for retirement.

For this study, we used data from the 2014 wave of the UAS, focusing on participants across a wide age range: 18 to 75+. The primary outcome variable of interest was retirement preparedness, measured using a continuous scale, with higher scores indicating greater financial readiness for retirement. This measure captured participants’ perceptions of their financial stability and preparedness as they approach or enter retirement, taking into account factors such as savings, pension, and income stability. In addition to retirement preparedness, participants provided demographic information, including their sex, age, marital status, country of birth (U.S.-born vs. non-U.S.-born), and current employment status. These variables were included as covariates to account for potential confounding effects on retirement preparedness.

Linear regression models were employed to examine the relationship between educational attainment (measured by years of schooling) and the retirement preparedness score, while adjusting for key demographic factors such as race, ethnicity, age, sex, employment status, marital status, and nativity. Two models were specified for the analysis: **Model 1** included educational attainment, race, ethnicity, and other control variables, without interaction terms. **Model 2** incorporated interaction terms between race/ethnicity and years of education to assess whether the relationship between educational attainment and retirement preparedness varied by race and ethnicity. Results were reported as beta coefficients, along with p-values and 95% confidence intervals (CIs). This analytical approach allowed for a detailed assessment of potential differences in how educational attainment impacts retirement preparedness across racial and ethnic groups, with a particular focus on the Minorities’ Diminished Returns (MDRs) theory [40–48]. According to MDRs, the effects of educational attainment on outcomes like retirement preparedness may be less substantial for Latino and Black individuals compared to non-Latino Whites [22, 23, 27, 29, 31, 34, 49].

### 2.2. Ethical Considerations

All participants provided consent as a part of their enrollment in the UAS panel. The study protocol was approved by the University of Southern California’s Institutional Review Board (IRB). All data were stored and analyzed anonymously.

## 3. Results

Table 1 shows the descriptive statistics for the sample (n = 3,602). The majority of participants were White (89.5%, n = 3,224), with 10.5% identifying as Black (n = 378). In terms of ethnicity, 92.0% of the sample identified as non-Latino (n = 3,314), while 8.0% were Latino (n = 288). Regarding U.S. birth status, 95.3% of the participants were non-immigrants (n = 3,434), with 4.7% being immigrants (n = 168). In terms of gender, 58.8% were women (n = 2,119), and 41.2% were men (n = 1,483). The majority of participants were employed (72.4%, n = 2,609), while 27.6% were not working (n = 993). Additionally, 58.0% of the sample reported being married (n = 2,089), with 42.0% not married (n = 1,512). The mean age of the participants was 43.40 years (SD = 12.04), and the average years of education was 11.02 years (SD = 2.22).

Table 2 shows the summary of linear regression without the interaction term. This model showed that each additional year of schooling was associated with a significant increase in retirement preparedness (B = 0.054, SE = 0.004, 95% CI: 0.047, 0.061, p < 0.001). Being Latino was significantly associated with lower retirement preparedness (B = −0.112, SE = 0.029, 95% CI: −0.169, −0.055, p < 0.001). Similarly, being Black was also significantly associated with lower retirement preparedness (B = −0.151, SE = 0.025, 95% CI: −0.201, −0.102, p < 0.001). Age had a significant positive association with retirement preparedness (B = 0.009, SE = 0.001, 95% CI: 0.008, 0.010, p < 0.001). Being female was negatively associated with retirement preparedness (B = −0.048, SE = 0.016, 95% CI: −0.078, −0.017, p = 0.002). Working was associated with a significant increase in retirement preparedness (B = 0.159, SE = 0.018, 95% CI: 0.124, 0.193, p < 0.001), and being married was also positively associated with higher retirement preparedness (B = 0.089, SE = 0.016, 95% CI: 0.058, 0.120, p < 0.001). Being born in the U.S. was not significantly related to retirement preparedness (B = 0.033, SE = 0.037, 95% CI: −0.040, 0.105, p = 0.377).

Table 3 shows the linear regression model with interaction terms examined retirement preparedness as the outcome. Each additional year of schooling significantly increased retirement preparedness (B = 0.101, SE = 0.013, 95% CI: 0.075, 0.127, p < 0.001). The interaction between education and being Black showed a significant negative association with retirement preparedness (B = −0.040, SE = 0.011, 95% CI: −0.063, −0.018, p < 0.001), as did the interaction between education and being Latino (B = −0.031, SE = 0.011, 95% CI: −0.053, −0.009, p = 0.006). Age had a significant positive association with retirement preparedness (B = 0.009, SE = 0.001, 95% CI: 0.008, 0.010, p < 0.001). Being female was negatively associated with retirement preparedness (B = −0.047, SE = 0.016, 95% CI: −0.078, −0.017, p = 0.002). Being born in the U.S. was not significantly associated with retirement preparedness (B = 0.038, SE = 0.037, 95% CI: −0.035, 0.111, p = 0.305). Working was associated with a significant increase in retirement preparedness (B = 0.160, SE = 0.018, 95% CI: 0.125, 0.194, p < 0.001), as was being married (B = 0.089, SE = 0.016, 95% CI: 0.058, 0.120, p < 0.001).

## 4. Discussion

The primary aim of this study was to examine the relationship between educational attainment—measured in years of schooling—and retirement preparedness in a national sample of U.S. adults. Specifically, we aimed to explore whether this relationship varied by race and ethnicity, utilizing the framework of Minorities’ Diminished Returns (MDRs). MDRs theory posits that the positive effects of educational attainment and other socioeconomic resources are often less pronounced for racial and ethnic minorities compared to non-Latino Whites. This diminished return is largely attributed to the marginalization of these groups, who often receive lower-quality education and are more likely to work in less favorable occupations. By investigating the impact of education on retirement preparedness among Black, Latino, and non-Latino White individuals, we sought to determine whether disparities in retirement outcomes could be explained by these differential returns on education across racial and ethnic groups.

Our findings indicate that, overall, higher educational attainment is significantly associated with greater retirement preparedness, underscoring the critical role that years of schooling play in determining financial security later in life. However, the results also provide strong support for the Minorities’ Diminished Returns (MDRs) framework [34], which reflects the structural inequalities present in U.S. society that shape the experiences of diverse groups. While education improved retirement preparedness across all racial and ethnic groups, the effects of educational attainment were significantly weaker for both Black and Latino individuals compared to non-Latino Whites. Even with similar levels of education, Black and Latino participants were less likely to report being financially prepared for retirement. These findings highlight the persistent structural barriers faced by racial and ethnic minorities, even when they achieve higher levels of education.

The results of this study support the Minorities’ Diminished Returns (MDRs) theory [34], which posits that the benefits of education and other socioeconomic resources are not distributed equally across racial and ethnic groups. In the context of retirement preparedness, our findings show that while education is a critical determinant, its effects are less pronounced for Black and Latino individuals. This suggests that the structural advantages conferred by education, such as higher income, better job opportunities, and improved financial literacy, are not fully accessible to racial and ethnic minorities in the same way they are to non-Latino Whites [19–21]. This diminished effect of education aligns with previous research in other areas, such as health outcomes and wealth accumulation, where similar patterns have been observed.

Minorities’ Diminished Returns (MDRs) refer to the phenomenon where the benefits of socioeconomic factors, like education, wealth, and employment, are reduced for racial and ethnic minorities compared to non-minorities [50–61]. MDRs exist due to structural inequalities and systemic barriers that disproportionately affect marginalized groups. For example, even highly educated Black and Latino individuals may face discrimination in the labor market, leading to lower-paying jobs or fewer opportunities for career advancement compared to similarly educated non-Latino Whites [34]. Additionally, minorities often attend underfunded schools and live in communities with fewer resources, which may result in a lower quality of education, even when they achieve higher degrees. Historical and ongoing racism, residential segregation, and inequities in the tax system that funds public education further contribute to these disparities. As a result, the social and economic benefits typically associated with higher education do not fully materialize for these groups, as seen in their reduced retirement preparedness.

### 4.1. Implications

These findings have significant implications for both policy and practice. First, they suggest that improving educational attainment alone is insufficient to ensure equitable outcomes for racial and ethnic minorities, particularly regarding financial security in retirement. Policymakers must address the structural barriers that limit the ability of minorities to translate their educational achievements into improved retirement preparedness. This could involve reforms in labor market practices, anti-discrimination laws, and targeted financial planning support for minorities. Additionally, the disparities highlighted by this study underscore the need for broader investments in minority communities to ensure that the quality of education and access to resources such as financial literacy programs are on par with those available to non-Latino Whites.

There are several reasons why education may have weaker effects for racial and ethnic minorities. One key factor is the unequal distribution of resources in public schools, which are often funded by local property taxes [62]. This system disproportionately disadvantages minority communities, which tend to have lower property values and, consequently, less funding for schools [63]. As a result, even when minority students complete the same number of years of schooling as their White counterparts, they may not receive the same quality of education [64–68]. Furthermore, racial and ethnic minorities are more likely to face labor market discrimination, resulting in lower wages, fewer promotions, and less access to high-quality retirement benefits, even with equivalent educational credentials [69–74]. These factors may combine to weaken the relationship between education and retirement preparedness for minorities, as their education does not lead to the same financial outcomes as it does for non-Latino Whites.

### 4.2. Limitations

This study has several limitations. ***First***, the data used are cross-sectional, which limits our ability to draw causal inferences about the relationship between education and retirement preparedness. Longitudinal studies are needed to track how education influences financial outcomes over the life course and into retirement. ***Second***, while we controlled for several key demographic variables, there may be unmeasured confounding factors, such as early-life socioeconomic status, access to high-quality education, and financial literacy, that could influence the relationship between education and retirement preparedness. Additionally, the study only included self-reported measures of retirement preparedness, which may not capture the full range of financial readiness for retirement. Future studies should incorporate objective measures, such as savings and pension amounts, to provide a more comprehensive assessment.

## 5. Conclusion

In conclusion, this study provides compelling evidence that, while educational attainment is a key determinant of retirement preparedness among U.S. adults, its benefits are not equally distributed across racial and ethnic groups. Minoritized groups, such as Black and Latino individuals, experience weaker returns on their education compared to their more socially privileged counterparts. These diminished returns on educational attainment in terms of retirement preparedness are likely driven by structural inequalities and systemic barriers such as discrimination. These findings underscore the need for policies that go beyond simply promoting educational attainment and address the broader societal factors that limit racial and ethnic minorities from fully benefiting from their educational achievements. Efforts to reduce disparities in retirement preparedness must focus on dismantling structural barriers and enhancing the quality of education, job opportunities, and financial resources available to minority communities.

## Figures and Tables

**Table 1.** Descriptive Data Overall (n = 3,602)

	N	%
**Race**		
White	3,224	89.5
Black	378	10.5
**Ethnicity**		
Non-Latino	3,314	92.0
Latino	288	8.0
**US Born Status**		
immigrant	168	4.7
Non-immigrant	3,434	95.3
**Sex**		
Men	1,483	41.2
Women	2,119	58.8
**Working**		
No	993	27.6
Yes	2,609	72.4
**Married**		
No	1,512	42.0
Yes	2,089	58.0
	Mean	SD
**Age (Year)**	43.40	12.04
**Education (Year)**	11.02	2.22
**Retirement Preparedness**	.87	.50

**Table 2.** Summary of Linear Regression Without Interaction Terms for Retirement Preparedness

Variable	B	SE	Beta	95%	CI	p
Ethnicity (Latino)	−0.112	0.029	−0.061	−0.169	−0.055	<0.001
Race (Black)	−0.151	0.025	−0.093	−0.201	−0.102	<0.001
Age (Year)	0.009	0.001	0.217	0.008	0.010	<0.001
Gender (Female)	−0.048	0.016	−0.047	−0.078	−0.017	0.002
Years of Schooling	0.054	0.004	0.239	0.047	0.061	<0.001
US Born	0.033	0.037	0.014	−0.040	0.105	0.377
Working	0.159	0.018	0.142	0.124	0.193	<0.001
Married	0.089	0.016	0.088	0.058	0.120	<0.001

Note: Dependent Variable = Retirement Preparedness

**Table 3.** Summary of Linear Regression with Interaction Terms for Retirement Preparedness

Variable	B	SE	Beta	95%	CI	p
Ethnicity (Latino)	0.221	0.125	0.120	−0.024	0.465	0.077
Race (Black)	0.274	0.123	0.168	0.032	0.516	0.026
Age (Year)	0.009	0.001	0.219	0.008	0.010	<0.001
Gender (Female)	−0.047	0.016	−0.047	−0.078	−0.017	0.002
Years of Schooling	0.101	0.013	0.448	0.075	0.127	<0.001
US Born	0.038	0.037	0.016	−0.035	0.111	0.305
Working	0.160	0.018	0.143	0.125	0.194	<0.001
Married	0.089	0.016	0.088	0.058	0.120	<0.001
Education × Black	−0.040	0.011	−0.317	−0.063	−0.018	<0.001
Education × Latino	−0.031	0.011	−0.183	−0.053	−0.009	0.006

Note: Dependent Variable = Retirement Preparedness
